# Therapeutic Effect and Mechanism of Bushen-Jianpi-Jiedu Decoction Combined with Chemotherapeutic Drugs on Postoperative Colorectal Cancer

**DOI:** 10.3389/fphar.2021.524663

**Published:** 2021-03-22

**Authors:** Meng-Die Yang, Wen-Jun Zhou, Xiao-Le Chen, Jian Chen, Qing Ji, Qi Li, Wen-Hai Wang, Shi-Bing Su

**Affiliations:** ^1^ Research Center for Traditional Chinese Medicine Complexity System, Institute of Interdisciplinary Integrative Medicine Research, Shanghai University of Traditional Chinese Medicine, Shanghai, China; ^2^ Shanghai TCM-Integrated Hospital, Shanghai University of Traditional Chinese Medicine, Shanghai, China; ^3^ Department of Vascular Disease, Shanghai TCM-Integrated Institute of Vascular Disease, Shanghai, China; ^4^ Department of Oncology, Shuguang Hospital Affiliated to Shanghai University of Traditional Chinese Medicine, Shanghai, China; ^5^ Department of Oncology, Shanghai Baoshan Hospital of Integrated Traditional Chinese and Western Medicine, Shanghai, China

**Keywords:** BSJPJDD, therapeutic effect, post operation, colorectal cancer, mechanism

## Abstract

There is a lack of effective therapeutic drugs in patients with postoperative colorectal cancer (PCRC). This study aimed to investigate the therapeutic effect and mechanisms of Bushen-Jianpi-Jiedu decoction (BSJPJDD) combined with chemotherapeutic drugs (oxaliplatin) on PCRC with liver and kidney yin deficiency and spleen deficiency syndrome (LKYD-SDS) through the therapeutic evaluation of clinical therapy and the integrative analysis of network pharmacology, RNA-seq and label-free data, and experiment verification *in vitro*. In clinical therapy, the median progression-free survival (PFS) and Karnofsky performance score (KPS) were increased in PCRC patients by the aqueous extract of BSJPJDD combined with oxaliplatin treatment for three months, compared to oxaliplatin alone (*p* < 0.05). The integrative analysis showed that 559 differentially expressed genes (DEGs) and 11 differentially expressed proteins (DEPs) were regulated by BSJPJDD, among which seven bioactive compounds through 39 potential targets were involved in the regulation of multiple signaling pathways including MAPK, PI3K-Akt, and HIF-1, etc. In the experimental verification, an ELISA assay showed that plasma ZEB2, CAT, and KRT78 were decreased, and IL-1Α, CD5L, FBLN5, EGF, and KRT78 were increased in comparison to the above (*p* < 0.05). Furthermore, the SW620 cell viability was inhibited and the expressions of MAPK and the p-ERK/ERK ratio were significantly downregulated by the aqueous extract of BSJPJDD combined with oxaliplatin treatment, compared with oxaliplatin treatment alone (*p* < 0.05). These data suggested that BSJPJDD combined with oxaliplatin prolongs the survival and improves Karnofsky performance status of PCRC patients with LKYD-SDS, and may be associated with the regulation of multiple signaling pathways.

## Introduction

Colorectal cancer (CRC), one of the most common cancers in the world, has become the third and second leading cause of cancer death among men and women, respectively (Barzi et al., 2017; Chen et al., 2017; Bos et al., 2018). Most patients of CRC are diagnosed at clinical stages II-III (Bos et al., 2018). As the main adjuvant treatment after CRC surgery, chemotherapy still plays a role in CRC treatment (Di Costanzo et al., 2003). However, the use of chemotherapy alone is insufficient, as there are adverse reactions to chemotherapy; the poor tolerance of patients and chemotherapy resistance (Panczyk, 2014; Li et al., 2019), therefore, an adjuvant therapy of postoperative chemotherapy is needed. The efficacy of drugs is usually evaluated by KPS which is a scoring standard for the functional status of the body. The higher the score, the better health condition, and the more the body can tolerate the side effects of treatment with a higher quality of life. It has been reported that traditional Chinese medicine (TCM) combined with chemotherapy can improve CRC treatment, through reducing postoperative ileus and urinary, improving the tolerance of patients and the complications and resistance of chemotherapy, remodeling the gut microbiota and the tumor microenvironment, suppressing thymidylate synthase, and preventing cancer recurrence and metastasis, etc. (Tan et al., 2008; Ye et al., 2015; Liu et al., 2017a; Sui et al., 2017; Liu et al., 2018a; Zhang et al., 2018; Lv et al., 2019; Hong et al., 2020).

In China, TCM is often used for the treatment of CRC (Su et al., 2012; Yang et al., 2019)^.^ In the clinical practice of TCM, treatment is usually based on TCM syndrome differentiation (Hu et al., 2018). TCM syndrome types in CRC have different metabolic and molecular characteristics (Ji et al., 2017; Wu et al., 2019b), and their treatments are according to the different indications. Previous research has showed that about 12% of CRC patients have liver and kidney yin deficiency and spleen deficiency syndrome (LKYD-SDS) (Zhang et al., 2013). The clinical manifestation of LKYD-SDS can be seen as hypochondriac and costal pain, weakness of the waist and knee, hot waves, night sweats, dry mouth and throat, fatigue, tinnitus, abdominal distention, loose stool, pale or red tongue, little or light fur, and a thin pulse. CRC patients with LKYD-SDS are often treated with the Bushen-Jianpi rule. It also has been reported that Bushen-Jianpi formula (BSJPF) can improve the survival time of liver cancer patients and regulate cell apoptosis *in vitro* (Cai et al., 2018). Moreover, the Jianpi-Jiedu formula (JPJDF) can improve the survival rate in patients with stage II and III CRC and inhibits tumorigenesis, metastasis, and angiogenesis through the mTOR/HIF-1alpha/VEGF pathway in CRC (Wu et al., 2019a).

The Bushen-Jianpi-Jiedu decoction (BSJPJDD) is one of the Bushen-Jianpi formulae, and consists of eight Chinese herbs. It contains *Astragalus membranaceus Fisch*. ex Bunge., *Rehmannia glutinosa* (Gaertn.) DC., *Atractylodes macrocephala* Koidz., *Cornus officinalis* Siebold & Zucc., *Dioscorea oppositifolia* L., *Sophora flavescens* Aiton., *Vitis quinquangularis* Rehd., and *Akebia trifoliata* (Thunb.) Koidz*.* However, the effects and molecular mechanisms of the aqueous extract of BSJPJDD combined with chemotherapeutic drugs (oxaliplatin) on CRC with LKYD-SDS are still unclear.

For many years, TCM has relied on the herbal formula, through a synergistic effect of various herbal medicines to achieve attenuation and efficiency in the treatment of complex diseases and syndromes. But it is difficult to analyze the effective mechanism of the formula by traditional pharmacological evaluations. As new fields in TCM studies, network pharmacology, transcriptomics, proteomics, and systems biology can be used for clarifying the effects and molecular mechanisms of TCM with a system perspective (Wu et al., 1997; Shi et al., 2014), it provides an approach for the further investigation of BSJPJDD and improves chemotherapy to treat CRC along with LKYD-SDS.

Therefore, this study investigated the effects and the pharmacological mechanisms of the aqueous extract of BSJPJDD combined with oxaliplatin on postoperative CRC (PCRC) with LKYD-SDS using survival analysis, integrative analysis with RNA-seq and label-free and network pharmacology analysis, and pharmacological experiment verification *in vitro*. Our study showed that BSJPJDD combined with oxaliplatin prolongs the survival and improves the life quality of PCRC patients with LKYD-SDS, and its pharmacological mechanisms may be involved in the comprehensive regulation of multi-compounds, multi-targets, and multi-pathways.

## Materials and Methods

### Reagents

Tetrazolium compound 3-(4,5-dimenthyl-2-yl)-5-(3carboxymethoxyphenyl)-2- (4sulfophenyl)-2H-tetrazolium MTS (lot# 313103) was obtained from Promega (United States). CD 3, CD 4, CD 8, and NK cell assay kits were obtained from BD Biosciences Pharmingen (United States). Oxaliplatin (bzl800003) was purchased from Aosaikang (Nanjing, China) Antibodies. The primary antibodies against ERK (lot# 4695), p-ERK (lot# 4370), p38 (lot# 9212), p-p38 (lot# 4511), JNK (lot# 9252), and p-JNK (lot# 4668) from Cell Signaling Technology (United States) and the secondary antibody against GAPDH (lot# AF0911) from Affinity Biosciences (United States) were used.

### Bushen-Jianpi-Jiedu Decoction Preparation

The herbal composition of BSJPJDD is shown in Table 1. All herbs were purchased from ShuGuang Hospital, Shanghai University of TCM and soaked in 2 L of water for 30 min, boiled for 30 min, then filtered three times. Finally, a concentration of 5.6 g/ml of aqueous extracts were made. HPLC-MS MRM chromatograms of the aqueous extracts of BSJPDD are shown in Supplementary Figure S1. The concentration of morroniside and loganin in BSJPJDD were 0.092 μg/ml (Supplementary Figure S1C) and 0.231 mg/ml (Supplementary Figure S1D), respectively. The reference standard of morroniside and loganin were purchased from the Shanghai Research Center of Chinese Medicine Standardization. The mobile phases comprised eluent A (0.1% formic acid) and eluent B (methanol). The gradient flow was as follows: 0–5 min, 30% of B; 5–40 min, 30–90% of B; 40–45 min, 90–100% of B; 45–50 min, 100% of B. The analysis was performed at a flow rate of 1.0 ml/min. The injection volume was 10 μL.

**TABLE 1:** The herbal composition of BSJPJDD.

Herbs	Dose (g)
*Astragalus membranaceus Fisch. ex Bunge*	18
*Rehmannia glutinosa (Gaertn.) DC.*	15
*Atractylodes macrocephala Koidz*	15
*Cornus officinalis Siebold* & *Zucc*	9
*Dioscorea oppositifolia* L.	9
*Sophora flavescens Aiton*	9
*Vitis quinquangularis Rehd*	30
*Akebia trifoliata (Thunb.) Koidz*	30

### Patient Characteristics and Treatments

A total of 149 patients with CRC were recruited from March 2014 to February 2018 at the Shanghai University of TCM Shu Guang Hospital. Patients were divided into a BSJPJDD group and oxaliplatin group (control group). The oxaliplatin group was treated following chemotherapy plans including FOLFIRI (Irinotecan 180 mg/m^2^, Day 1 + Leucovorin 400 mg/m^2^, Day 1 + Fluorouracil 400 mg/m^2^, Day 1 + Fluorouracil 2,400 mg/m^2^ until 48 h), XELOX (Oxaliplatin 130 mg/m^2^, Day 1 + Xeloda 1,000 mg/m^2^, bid for 14 days), FOLFOX4 (Oxaliplatin 85 mg/m^2^, Day 1 + LV 200 mg/m^2^, Day 1 and Day 2 + 5-FU 400 mg/m^2^, Day 1 and Day 2), and FOLFOX6 (Oxaliplatin 85 mg/m^2^, Day 1 + leucovorin 400 mg/m^2^, Day 1 + fluorouracil 400 mg/m^2^, Day 1 + fluorouracil 2,400 mg/m^2^ until 48 h). The BSJPDD group was treated with the aqueous extract of BSJPDD (5.6 g/ml, once a day) based on the treatment of the above chemotherapy plans. The all of patients underwent ≥12 weeks of treatments.

The inclusion criteria and the exclusion criteria are shown in Table 2. Additional information regarding this study includes the following: clinical trials, entitled Study of TCM Syndrome of Hepatocellular Carcinoma and Colorectal Cancer Based on System Science (govIdentifieris NCT03189992), and retrospectively registered in June 16, 2017. All experimental protocols were approved by the IRB of the Shuguang Hospital affiliated with the Shanghai University of Traditional Chinese Medicine (2014-345-41-01) in accordance with the Guidelines for Ethical Review of Drug Clinical Trials, the Declaration of Helsinki, the International Ethical Guidelines for Biomedical Research Involving Human Subjects (CIOMS: 2002).

**TABLE 2:** The inclusion and exclusion criteria of chemotherapy plans.

Inclusion criteria	Exclusion criteria
Over 18 years of age	Serious complications
PCRC patients with LKYD-SDS	With other serious diseases
Diagnosed by experts from shanghai university of TCM shu guang hospital and the diagnostic criteria was based on the guiding principles for clinical research on new drugs in TCM (Yang et al., 2019)	Incomplete medical records

### Total RNA Extractions and RNA-Seq Analysis

Total RNA in leukocytes from the PCRC patients with LKYD-SDS (n = 12), PCRC patients without LKYD-SDS (n = 9), the BSJPJDD group before treatment (n = 6), and the BSJPJDD group after treatment (n = 6) were isolated using TRIzol Reagent (Cat# 15596-018, Life Technologies, Carlsbad, CA, United States) following the manufacturer’s instructions and checked for an RNA integrity number (RIN) to inspect RNA integration using an Agilent Bioanalyzer 2,100 (Agilent Technologies, Santa Clara, CA, United States). Qualified total RNA was further purified using an RNeasy Mini Kit (Cat# 74106, QIAGEN, GmBH, Germany) and an RNase-Free DNase Set (Cat# 79254, QIAGEN) and stored at −80°C. Quality control was carried out using the RIN number obtained from the Agilent Bioanalyzer 2,100 (Agilent Technologies). An RNA-seq analysis was conducted on each total RNA sample by Illumina RS-122-2401. The data were normalized using Trimmomatic. The differentially expressed genes (DEGs) were screened with fold changes ≥1.5 and *p* ≤ 0.05.

### Sample Preparation and Label-Free Analysis

Plasma samples from the PCRC patients with LKYD-SDS (n = 3) and PCRC patients without LKYD-SDS (n = 3) were ground to a fine powder in liquid nitrogen and suspended in STD buffer (4% SDS, 1 mmol/L DTT, 150 mmol/L TrisHCl, pH 8.0). The suspension was vortexed and incubated in boiling water for 5 min. The supernatant was obtained by centrifugation after ultrasonication. The protein concentration of each sample was determined using a 2D Quantification Kit (GE Healthcare, Buckinghamshire, United Kingdom). A total of 100 μg of each sample was denatured, reduced, and alkylated as described in the label-free protocol. Each sample was digested overnight with 0.1 μg/μL of trypsin solution at 37°C. The digested peptides were dried by vacuum centrifugation. The nine samples were pooled and vacuum-dried. The pooled sample was separated on a Poly-LC strong cation exchange column (4.6 mm × 100 mm) using a Nano HPLC System (GE Healthcare). Subsequently, the fractionated samples were analyzed by LC-MS/MS based on a Q-Exactive mass spectrometer (Thermo Finnigan, CA, United States). For peptide data analysis, raw mass data were processed using the Proteomics Tools (Abcam, Cambridge, United Kingdom) and normalized according to signal values. The differentially expressed proteins (DEPs) were identified with BRB-array Tools.

### Enzyme-Linked Immunosorbent Assay Test

Plasma samples of PCRC patients with LKYD-SDS treated with BSJPJDD (n = 58) and without BSJPJDD (n = 58) were used in the ELISA test. ZEB2, IL-1A, CAT, RECK, LCP1, CD5L, EGF, ALML5, HLA-A, FBLN-5, and KRT78 were tested using ELISA kits according to protocols (ml0202932, Shanghai Enzyme-linked Biotechnology Co., Ltd., Shanghai, China). Briefly, a 50 µl serum sample was diluted 1:1, then a 50 µl biotin-labeled antibody was added into the reaction hole, incubated at 37°C for 1 h, and then 80 µl of streptavidin-HRP was added, incubated at 37°C for 30 min, then 50 µl of substrates A and B were added, incubated at 37°C for 10 min. OD value was measured at 450 nm after adding 50 µl of termination solution.

### Network Pharmacology Analysis

The chemical compounds and their targets of BSJPJDD were identified using the TCM database at Taiwan (http://tcm.cmu.edu.tw/), TCM systems pharmacology (TCMSP) database (http://lsp.nwu.edu.cn/tcmsp.php), and PDTCM (http://cadd.gdhtcm.com:2180/PDTCM/index.php). The compounds were screened for oral bioavailability (OB) > 30% and drug-likeness (DL) > 0.18. The targets of PCRC were collected from GeneCards (https://www.genecards.org/) and the OMIM database (www.omim.org/); the screening criteria were liver cancer, highlights, and scores >25. The targets networks of BSJPJDD on CRC were constructed by the Cytoscape software (Version 3.2.0, National Institute of General Medical Sciences, Bethesda, MD, United States). Related parameters were calculated to explore significant nodes (Liu et al., 2017b). An evaluation of the network stabilization was carried out according to Liu et al. (Song et al., 2016). All of the enriched functions and pathways were obtained by OmicsBean (www.omicsbean.cn/).

### MTS Assay

An SW620 cell is a human colorectal adenocarcinoma cell line. It has been chosen in this study according to previous research reports (Hu et al., 2019; Limagne et al., 2019). The SW620 cell line was purchased from the Type Culture Collection of the Chinese Academy of Sciences (Shanghai, China), and the cells were cultured as previously described (Labianca et al., 2010). SW620 cells were cultured in a 96-well plate with a density of 5.0 × 10^3^ cells/well for 24 h, followed by incubation with the aqueous extract of BSJPJDD (1.56, 3.13, 6.25, 12.5, 25, 50, and 100 mg/ml), oxaliplatin (1.56, 3.13, 6.25, 12.5, 25, 50, and 100 μg/ml), and BSJPJDD (3.84 or 1.92 mg/ml) combined with oxaliplatin (1.56, 3.13, 6.25, 12.5, 25, 50, and 100 µg/ml) at 37°C for 24 h. Then 20 μl of CellTiter AQ solution (G3580, Promega) was added to each well. After 4 h, the absorbance at 490 nm was measured, and the cell viability was calculated. The combination indices (CIs) were analyzed using the Compusyn software (ComboSyn, Paramus, United States). The CI value <1 denotes a synergistic effect, the CI value = 1 denotes an additive effect, and the CI value >1 denotes an antagonistic effect (Chou and Talalay, 2010).

### Western Blot Assay

SW620 cells were cultured in a 6-well plate with a density of 1.0 × 10^6^ cells/well for 24 h followed by incubation with 3.84 mg/mL of BSJPDD extracts and 15.8 µg/ml of oxaliplatin for 48 h. Then, the cells were collected and assayed as described in previous literature. The primary antibodies including ERK, p-ERK, p38, p-p38, JNK, and *p*-JNK (all of 1:1,000) and secondary antibody GAPDH (1:5,000) were used. Gray value of the Western blot results was calculated by the one-way analysis of variance (ANOVA), performed by ImageJ 1.8.0. *p* < 0.05 was considered significant. The assays were repeated at least three times.

### Statistical Analysis

The one-way analysis of variance (ANOVA), rank-sum test, Kaplan-Meier curves, univariate analysis, multivariate Cox regression analysis, and Karnofsky performance score (KPS) analysis were performed (SPSS 18.0 software). *p* < 0.05 was considered significant.

## Results

### Patient Characteristics, Survival, and Karnofsky Performance Score Analysis

**FIGURE 1:**
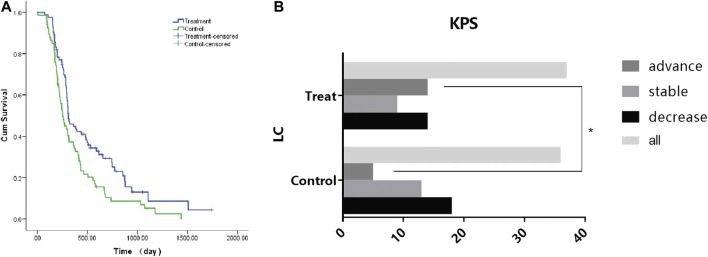
Survival and KPS analysis in CRC treated with BSJPJDD (n = 3) snd without BSJPJDD (n = 66). **(A)** Survival analysis, **(B)** KPS *p < 0.05, BSJPJDD group vs control group.

In this study, 83 (55.7%) CRC patients with LKYD-SDS were treated with the aqueous extract of BSJPJDD combined with oxaliplatin. The patient characteristics and baseline are listed in Table 3. As shown in Figure 1A, the survival analysis revealed that the median progression-free survival (PFS) of the BSJPJDD group was longer than those of the control group (*p* = 0.008). As shown in Figure 1B, the KPS in advanced stage in the BSJPJDD group was significantly increased compared to the control group (*p* < 0.05). These results indicated that BSJPJDD may increase the efficacy of oxaliplatin, improve the survival time and Karnofsky performance status of PCRC patients with LKYD-SDS.

**TABLE 3:** Characteristics and baseline data of patients with PCRC.

Characteristics	BSJPJDD (N = 83) n (%)	Control (N = 66) n (%)	*p*
Age	62.85 ± 9.26	60.95 ± 9.88	0.137
Gender			0.824
Male	25 (30.12)	21 (31.82)	
Female	58 (69.88)	45 (68.18)	
Pathological differentiation			0.944
Middle-high	65 (78.31)	52 (78.79)	
Low	18 (21.69)	14 (21.21)	
Immune cells marker			
CD3 (%)	61.53 ± 11.16	63.49 ± 13.2	0.904
CD4 (%)	34.63 ± 10.07	38.59 ± 11.36	0.166
CD8 (%)	32.2 ± 11.67	30.18 ± 10.93	0.440
CD4/CD8 (%)	2.2 ± 5.83	1.54 ± 0.94	0.403
NK (%)	20.85 ± 12.05	19.98 ± 10.22	0.742

### Differentially Expressed Genes and Network Pharmacology Analysis of Bushen-Jianpi-Jiedu Decoction in PCRC Patients with Liver and Kidney Yin Deficiency and Spleen Deficiency Syndrome

**FIGURE 2:**
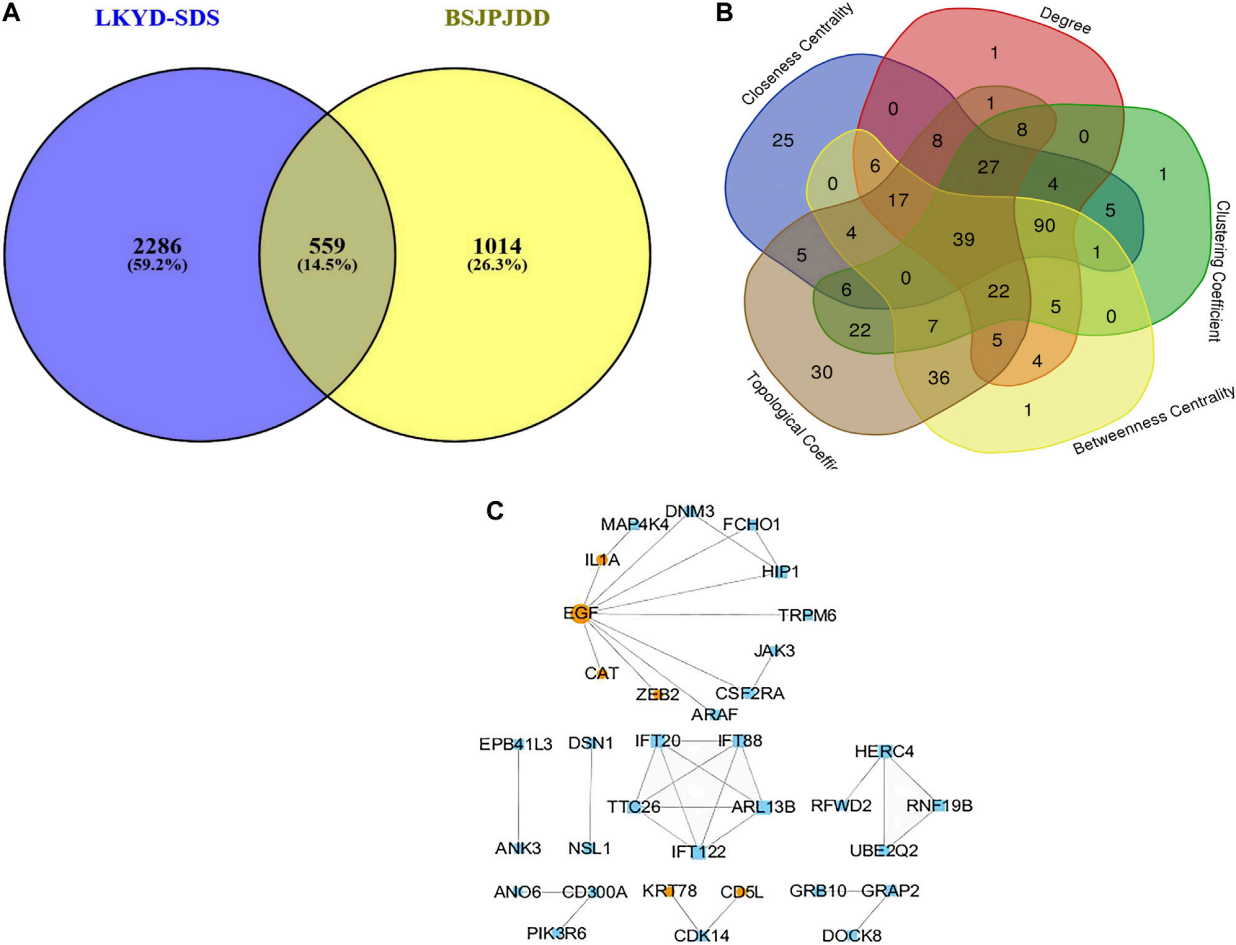
Venn analysis and network pharmacology analysis of BSJPJDD in CRC patients with LKYD-SDS. **(A)** Venn Analysis, **(B)** Network Topological analysis, **(C)** targets networks BSJPJDD.

**FIGURE 3:**
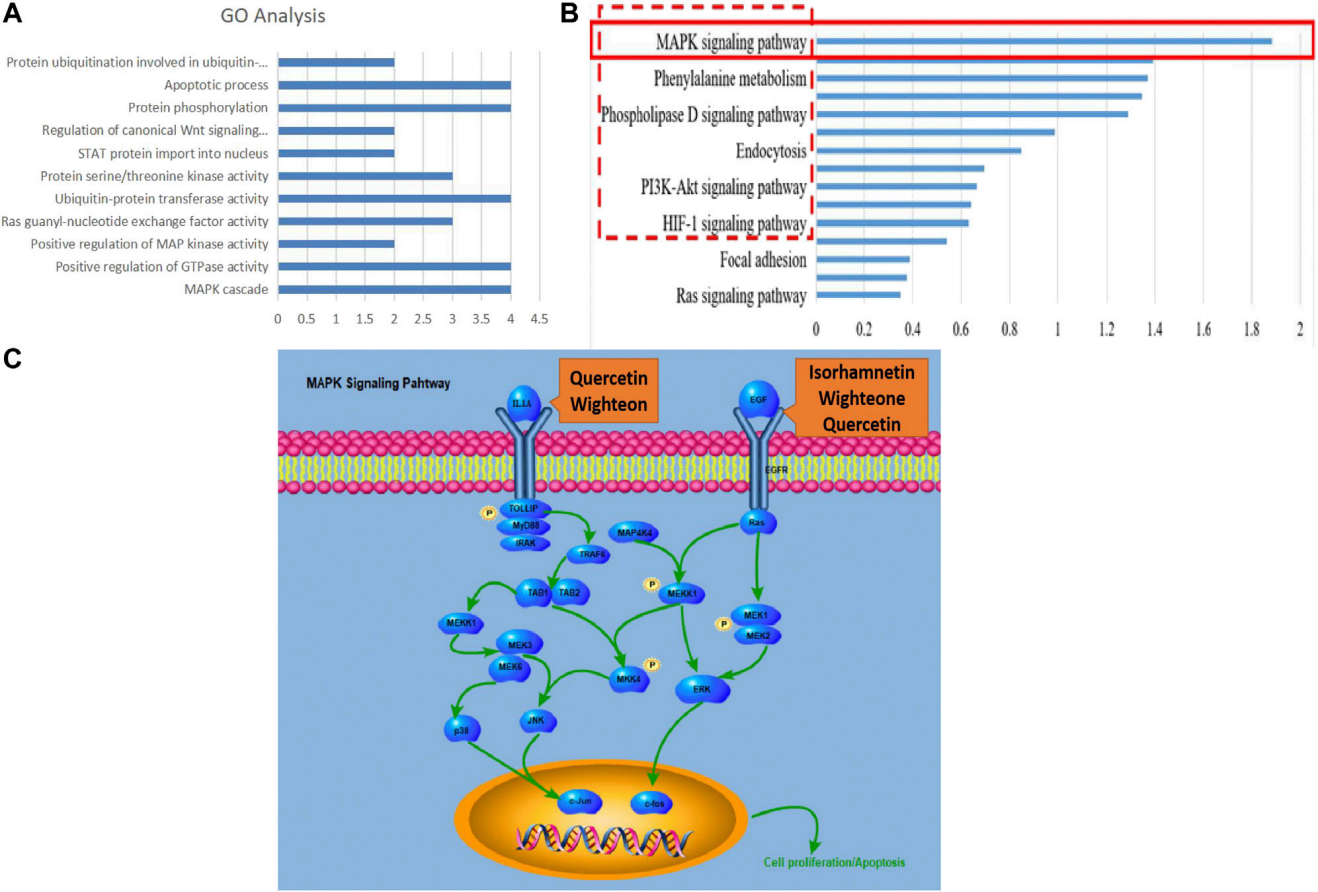
GO and pathway enrichment analysis and components-targets pathways process of BSJPJDD therapy. **(A)** enriched GO, **(B)** enriched signaling pathways, **(C)** components -targets-pathways-process.

The expressions of mRNA were detected by RNA-seq analysis. A total of 2,845 DEGs in PCRC patients with and without LKYD-SDS and 1,573 DEGs in PCRC patients treated with and without BSJPJDD were found. Venn analysis shows that there were 559 crossed DEGs (Figure 2A), indicating that there are DEGs of BSJPJDD in PCRC patients with LKYD-SDS. A BSJPJDD network consisted of 559 DEGs and 11 DEPs. It was constructed to screen the major potential targets of BSJPJDD on PCRC with LKYD-SDS treatment. After a comprehensive evaluation by the topological parameters including topological coefficient, clustering coefficient, degree, closeness centrality, and betweenness centrality, 39 nodes were retained (Figure 2B). Based on these 39 nodes, a targets network of BSJPJDD was constructed, as shown in Figure 2C. Moreover, the major functions and signaling pathways of BSJPJDD on PCRC with LKYD-SDS treatment were found by the enrichment analysis of GO (Figure 3A) and pathway (Figure 3B). It shows that BSJPJDD was involved in the regulation of multiple functions including MAPK cascade, positive regulation of MAP kinase activity, protein binding, Ras guanyl-nucleotide exchange factor activity, regulation of canonical Wnt signaling pathway, etc., and multiple signaling pathways, including MAPK, PI3K-Akt, and HIF-1, etc. Furthermore, it was found that seven bioactive compounds (isorhamnetin, kaempferol, 8-Isopentenyl-kaempferol, norartocarpetin, wighteone, quercetin, and beta-sitosterol) were found in the BSJPJDD, among which quercetin and wighteone targeted IL-1A regulating P38, JNK, and isorhamnetin, and wighteone and quercetin targeted EGF regulating JNK and ERK in the MAPK pathway (Figure 3C), which further indicated how the bioactive compounds of BSJPJDD are involved in the regulation of the these signal pathways through their targets.

### Effects of Bushen-Jianpi-Jiedu Decoction on the Plasma Protein Expressions in PCRC Patients with Liver and Kidney Yin Deficiency and Spleen Deficiency Syndrome

**FIGURE 4:**
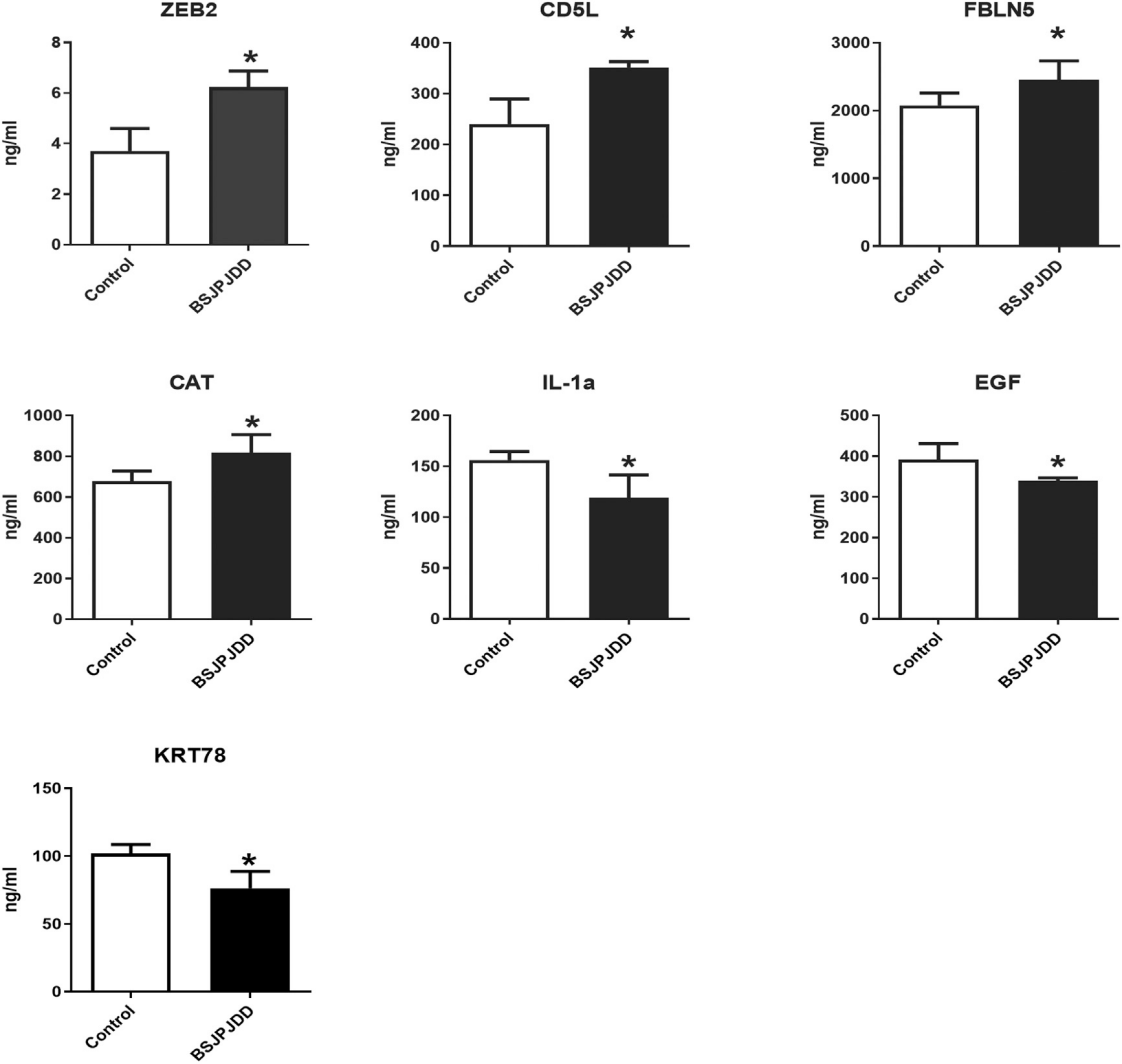
Plasma protein expressions in PCRC patients with LYKD-SDS treated with BSJPJDD (n = 58) and without BSJPJDD (n = 58). The validation tests were carried out by ELISA plasma ZEB2,CD5L,FBLN5, and CAT were up regulated,IL-1A, EGF and KRT78 were down-regulated by BSJPJDD *p < 0.05, BSJPJDD group vs control group.

The expressions of plasma proteins were detected by label-free analysis. Eleven DEPs were found in CRC patients with LKYD-SDS treated with and without BSJPJDD. Furthermore, the DEPs were verified by the ELISA assay. As shown in Figure 4, plasma ZEB2, CD5L, FBLN5, and CAT were significantly increased, IL-1Α, EGF, and KRT78 were significantly decreased in the BSJPJDD group compared to the control group (*p* < 0.05). It indicated that these plasma proteins may be potential indicators for assessing the effects of BSJPJDD on PCRC with LKYD-SDS treatment.

### Synergistic Effects of Bushen-Jianpi-Jiedu Decoction Combined with Oxaliplatin on SW620 Cell Viability

**FIGURE 5:**
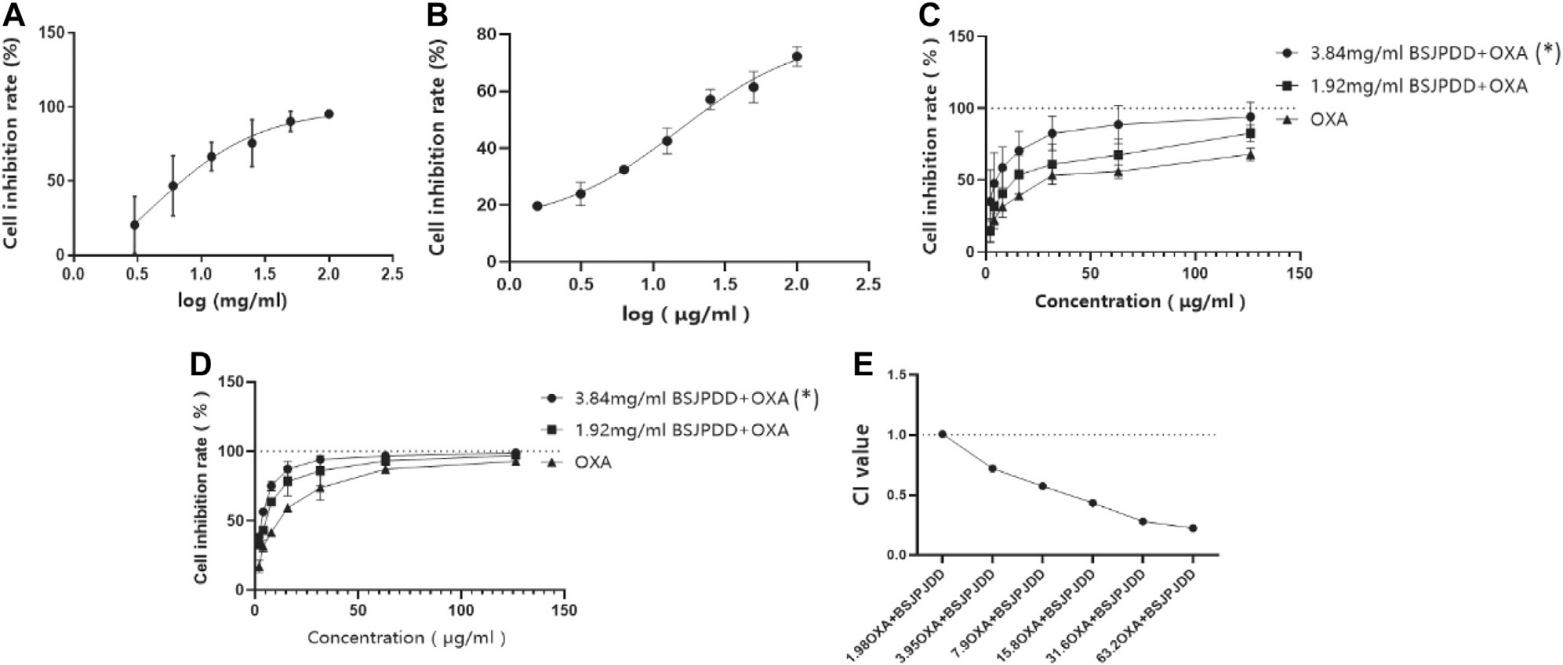
Effects of BSJPJDD and oxapilation on SW620 cells viability. **(A)** Treated with BSJPJDD, **(B)** treated with oxaliplatin, **(C)** BSJPJDD and oxaliplatin on SW620 cells viability for 24 h, **(D)** BSJPJDD and oxaliplatin on SW620 cells viability for 48 h, **(E)** synergistic effect analysis. Cell viability was measured by MTS assay. Values represent the mean ± SD from three independent experiments. OXA oxaliplatin *p < 0.05, BSJPJDD combined with oxaliplatin vs oxaliplatin.

The cell viability was detected by an MTS assay. As shown in Figures 5A,B, the cell viability in SW620 cells were inhibited by the aqueous extract of BSJPJDD (IC_50_: 3.84 mg/ml) and oxaliplatin (IC_50_: 15.8 μg/ml) treatments in a concentration-dependent manner, respectively. Moreover, the inhibition rate of the SW620 cells in the 3.84 and 1.92 mg/ml BSJPJDD-treated groups were higher than that in the oxaliplatin-treated group, and there was a significant difference between the 3.84 mg/ml BSJPJDD-treated group and the oxaliplatin-treated group (*p* < 0.001), but not in those of the 1.92 mg/ml BSJPJDD-treated group (*p* > 0.05) for 24 h or 48 h (Figures 5C,D). The CI values of the combination treatment were 1.01, 0.72, 0.58, 0.44, 0.28, and 0.22, respectively (Figure 5E). It indicated that BSJPJDD combined with oxaliplatin had a synergistic effect that can inhibit the viability of SW620 cells, and that 3.84 mg/ml of BSJPJDD and 15.8 µg/ml of oxaliplatin were suitable for further experiments of effective mechanisms.

### Synergistic Effects of BSJPJDD and Oxaliplatin on the MAPK Pathway in SW620 Cells

**FIGURE 6:**
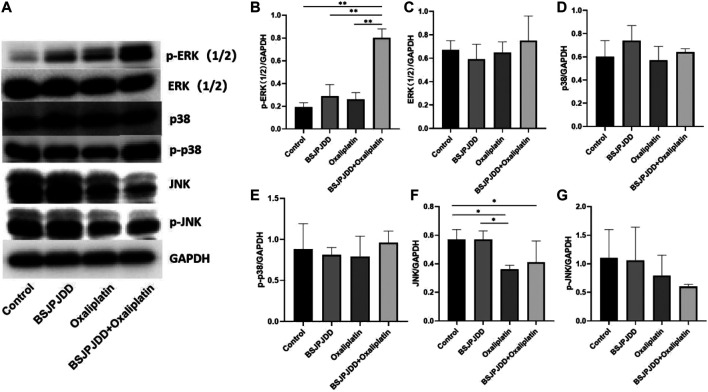
Expressions of MAPK pathway-related proteins treated by BSJPJDD and oxapilation in SW620 cells. **(A)** Fragments of Western Blot; **(B–G)** Expression of proteins (p-ERK,ERK,p38,p-p38,JNK,P-JNK) was quantified by ImageJ and normalized by GAPDH expression. Values represent the mean ± SD from three independent experiments *p < 0.05, **p < 0.001.

The expressions of ERK, p-ERK, p38, p-p38, JNK, and *p*-JNK of the MAPK pathway in SW620 cells were detected by Western blot assay. As shown in Figure 6, p-ERK1/2 ratio were significantly upregulated in the combination group (BSJPJDD + oxaliplatin) compared to each single treatment of BSJPJDD, oxaliplatin, or control (no treatment) (*p* < 0.001, Figure 6B) and JNK was significantly downregulated (*p* < 0.05, Figure 6F) in BSJPJDD combined with oxaliplatin compared to control alone. ERK1/2, p38, p-p38, and *p*-JNK had no obvious change (Figures 6C–E, G). These results indicated that the MAPK/ERK pathway was involved in the synergistic effects of BSJPJDD and oxaliplatin on SW620 cell viability.

## Discussion

Clinical relapse is a major reason for postoperative CRC-related deaths, about 33–50% of CRC patients relapse, and more than 90% of relapses occur during the first five years following surgery and at a particularly higher rate in the first two years (Binefa et al., 2014), therefore, an effective treatment is extremely important for PCRC. Currently, chemotherapy is still the main strategy for the treatment of PCRC (Li et al., 2013; Huang et al., 2019), which can prolong survival rate and reduce recurrence in patients. However, the tolerance of chemotherapeutic strength and quality of life in cancer patients during chemotherapy are important issues. Even though TCM has proven to be an effective therapy in CRC treatments (Zhang et al., 2018; Zhu et al., 2018), the effects and molecular mechanisms of TCM treatment combined with oxaliplatin on PCRC are still unclear.

This study investigates the therapeutic effect and mechanism of the aqueous extract of BSJPJDD combined with oxaliplatin (FOLFIRI, XELOX, FOLFOX4, and FOLFOX6) on PCRC with LKYD-SDS. After treatment for three months, PFS was significantly extended and KPS was significantly increased in PCRC patients treated with the aqueous extract of BSJPJDD combined with oxaliplatin compared with those of oxaliplatin alone (Figure 1). These results suggested that BSJPJDD is beneficial when used with oxaliplatin for PCRC patients with LKYD-SDS. Further research will demonstrate whether BSJPJDD combined with oxaliplatin treatment for 6–12 months is more effective than oxaliplatin alone for PCRC.

The treatment of cancer under the holistic or systemic view of TCM has the characteristics of overall regulation with multi-molecular, multi-targets, multi-function, and multi-pathways, which is consistent with the principle of systems biology and network pharmacology. It has been widely applied in effective mechanisms of TCM treatments (Zhang et al., 2013; Cai et al., 2018; Wu et al., 2019a) and also applied in the research of Chinese herbal formulae on CRC treatment (Liu et al., 2018b; Gong et al., 2019). This study further investigated the effective mechanisms of BSJPJDD by transcriptomics, proteomics, network pharmacology analysis, and experimental verification. A total of 559 DEGs and 11 DEPs were found to be involved in the regulation of BSJPJDD effects on PCRC patients with LKYD-SDS (Figure 2A). Our results (Figure 4) showed that ZEB2, CD5L, FBLN5, CAT, IL-1Α, EGF, and KRT78 may be the potential targets involved in the effective mechanisms of the aqueous extract of BSJPJDD combined with oxaliplatin for PCRC with LKYD-SDS treatment.

Moreover, according to the evaluation of primary network nodes and the shared signaling pathways of potential targets of BSJPJDD on PCRC with LKYD-SDS, it was found that BSJPJDD was involved in the regulation of multiple functions including MAPK cascade, positive regulation of MAP kinase activity, protein binding, Ras guanyl-nucleotide exchange factor activity, regulation of the canonical Wnt signaling pathway (Figure 3A), and multiple intracellular signaling, such as the MAPK, PI3K-Akt, and HIF-1 pathways (Figure 3B). Furthermore, seven bioactive compounds (isorhamnetin, kaempferol, 8-Isopentenyl-kaempferol, norartocarpetin, wighteone, quercetin, and beta-sitosterol) were found in the BSJPJDD, among which quercetin and wighteone targeted IL-1A regulating MEKK1, P38, MEK3, TAK1, and isorhamnetin, and wighteone and quercetin targeted EGF regulating JNK and ERK in the MAPK pathway (Figure 3E), indicating that these compounds of BSJPJDD might activate multiple pathways to inhibit tumor progression, increase survival, and improve life quality in PCRC patients.

However, distinguishing inhibitory effects from action effects is difficult in network pharmacology. Moreover, this type of analysis is susceptible to influence by prediction tools. Thus, it is essential to validate the predictions. The cell viability assay is commonly used to evaluate the efficacy of anti-tumor drugs. In this study, 3.84 mg/mL of BSJPJDD aqueous extract and 15.8 μg/mL of oxaliplatin significantly inhibited the viability of SW620 cells and its CI value was less than 1 (Figure 5). These results showed that there is a synergistic effect of the BSJPJDD aqueous extract and oxaliplatin on inhibiting SW260 cell proliferation.

The MAPK pathway, with three sub-pathways of p38 MAPK, ERK-1/2, and JNK, is an important signaling system that mediates cellular responses, and is involved in many physiological processes, such as cell growth, development, division, and death (Sun et al., 2015). Moreover, it is also an important signaling pathway in the process of the development and death of cancer (Kumar Pandurangan et al., 2018). Recently, it has been reported the MAPK pathway was involved in the regulation of CRC treated by Chinese herbal formulae (Chang et al., 2018). This study found the synergistic effect of BSJPJDD aqueous extract and oxaliplatin.

## Conclusion

**FIGURE 7:**
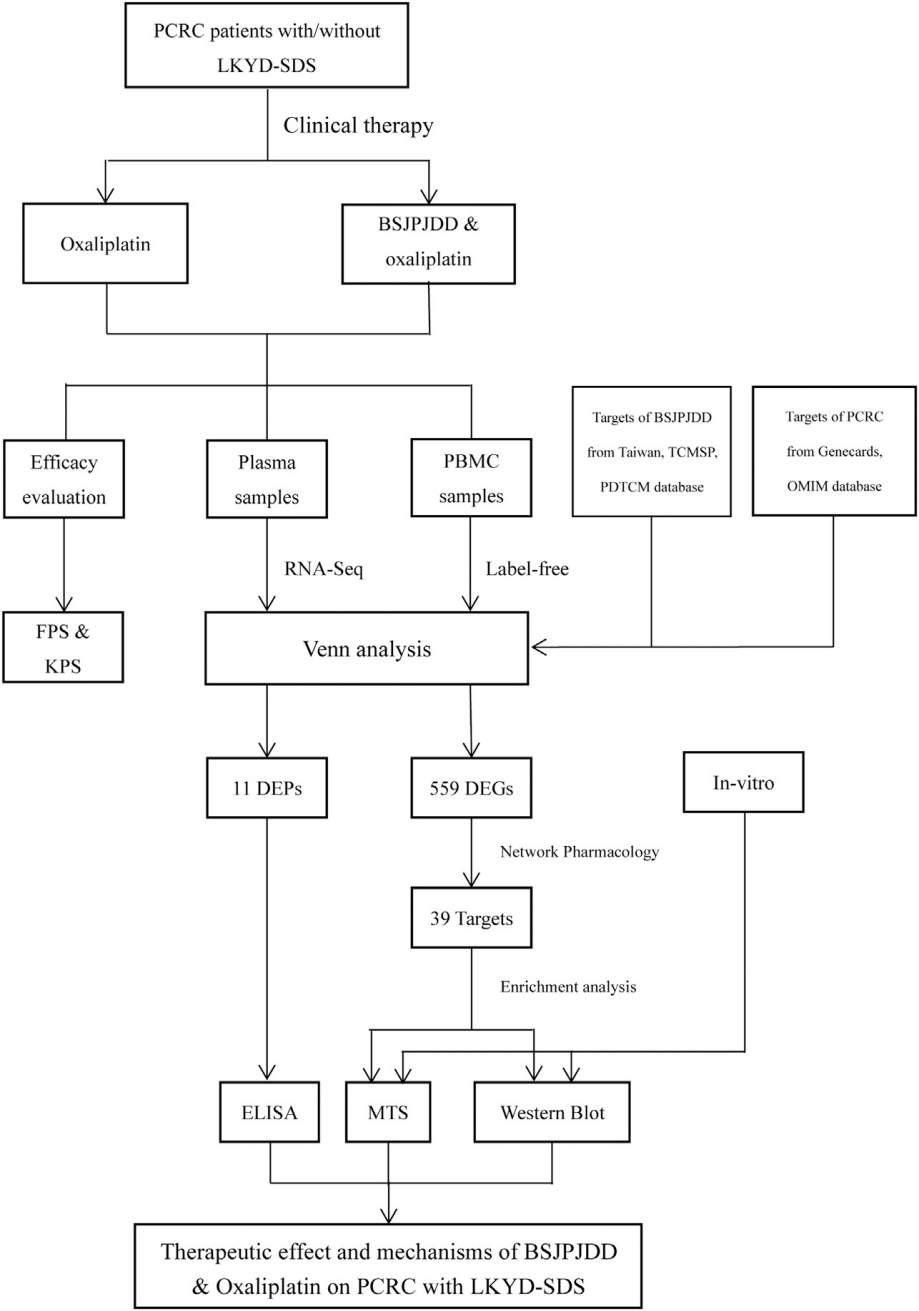
The graphical abstract of the entire circle.

In summary, the clinical efficacy of oxaliplatin including survival time and life quality in PCRC patients with LKYD-SDS were improved by the aqueous extract of BSJPJDD combined with oxaliplatin treatment. Its therapeutic mechanisms may be involved in the comprehensive regulation of BSJPJDD with multi-compounds multi-targets, and multi-efficacies, including the regulation of multiple plasma proteins such as the decreases of ZEB2, CAT, and KRT78 and the increases of IL-1Α, CD5L, FBLN5, EGF, and KRT78, and the regulation of multiple signaling pathways such as the downregulation of the MAPK pathway. The graphical abstract of the entire article is shown in Figure 7.

## Data Availability

The datasets presented in this study can be found in online repositories. The names of the repository/repositories and accession number(s) can be found below: NCBI BioProject, accession no: PRJNA682309.
