# Supplementary figures and images for: Therapeutic Effect and Mechanism of Bushen-Jianpi-Jiedu Decoction Combined with Chemotherapeutic Drugs on Postoperative Colorectal Cancer
Source: Front Pharmacol. 2021 Mar 22;12:524663. doi: 10.3389/fphar.2021.524663 (PMC8020259; doi:10.3389/fphar.2021.524663)

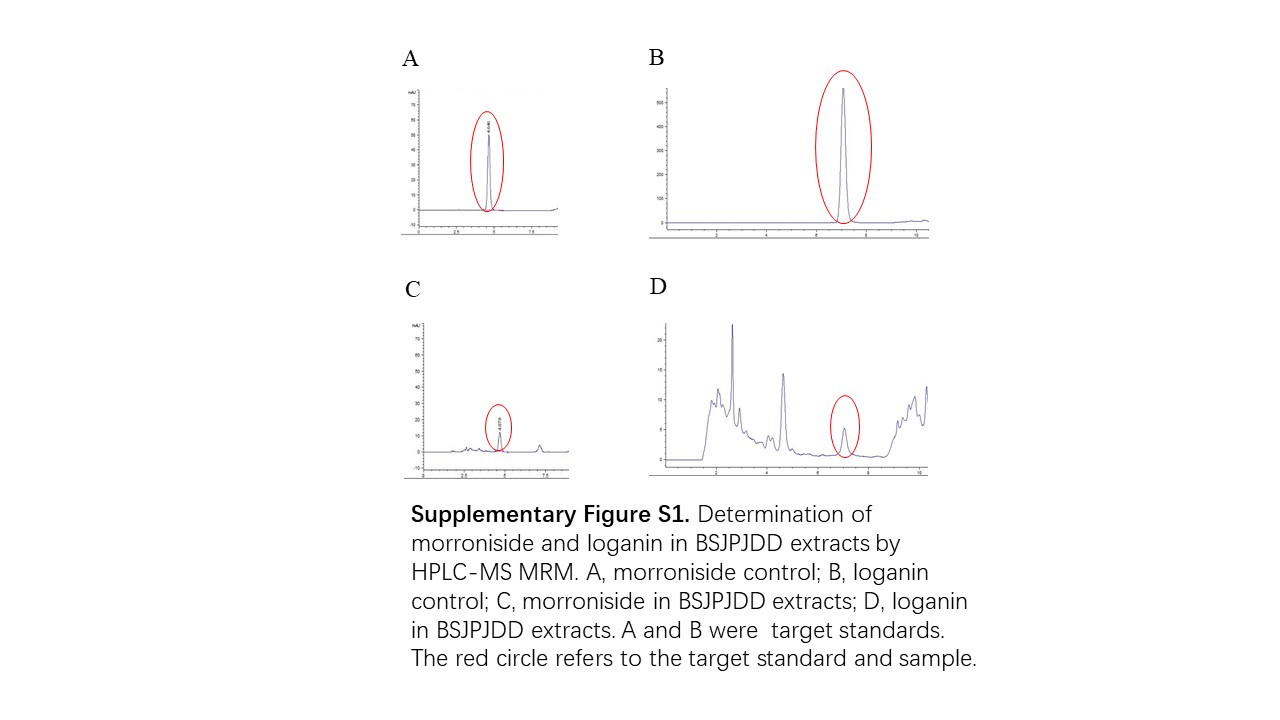

Supplement: Supplementary file 1 [file image1.jpg]
